# Chicken Mesenchymal Stem Cells and Their Applications: A Mini Review

**DOI:** 10.3390/ani11071883

**Published:** 2021-06-24

**Authors:** Andrea Svoradova, Vladimir Zmrhal, Eva Venusova, Petr Slama

**Affiliations:** Department of Animal Morphology, Physiology and Genetics, Faculty of AgriSciences, Mendel University in Brno, 613 00 Brno, Czech Republic; andrea.svoradova@mendelu.cz (A.S.); vladimir.zmrhal@mendelu.cz (V.Z.); eva.venusova@mendelu.cz (E.V.)

**Keywords:** chicken, mesenchymal stem cells, culture, disease, probiotics, applications

## Abstract

**Simple Summary:**

Mesenchymal stem cells (MSCs) are multipotent stem cells that are capable of differentiation into bone, muscle, fat, and closely related lineages and express unique and specific cell surface markers. They can be used as an avian culture model to better understand osteogenic, adipogenic, and myogenic pathways. Moreover, MSCs could also be used as a model to study various developmental and physiological processes in avian and other species. To obtain a comprehensive overview of this topic, the keywords “mesenchymal stem cells”, “chicken”, “disease”, “chicken dermatitis”, “viral infections in chicken”, and “antibiotics in chicken” were searched in WOS and PUBMED databases to obtain relevant information.

**Abstract:**

Mesenchymal stem cells (MSCs) are multipotent progenitor cells that adhere to plastic; express the specific markers CD29, CD44, CD73, CD90, and CD105; and produce cytokines and growth factors supporting and regulating hematopoiesis. MSCs have capacity for differentiating into osteocytes, chondrocytes, adipocytes, and myocytes. They are useful for research toward better understanding the pathogenic potential of the infectious bursal disease virus, mineralization during osteogenesis, and interactions between MSCs as a feeder layer to other cells. MSCs are also important for immunomodulatory cell therapy, can provide a suitable strategy model for coculture with pathogens causing dermatitis disorders in chickens, can be cultured in vitro with probiotics and prebiotics with a view to eliminate the feeding of antibiotic growth promoters, and offer cell-based meat production. Moreover, bone marrow-derived MSCs (BM-MSCs) in coculture with hematopoietic progenitor/stem cells (HPCs/HSCs) can support expansion and regulation of the hematopoiesis process using the 3D-culture system in future research in chickens. MSCs’ several advantages, including ready availability, strong proliferation, and immune modulatory properties make them a suitable model in the field of stem cell research. This review summarizes current knowledge about the general characterization of MSCs and their application in chicken as a model organism.

## 1. Introduction

As poultry meat production has increased dramatically in recent years, attention is now turning more to ensuring the high quality of that output [1]. Because genetic modification and selection for growth can cause skeletal disorders or muscle degeneration, and thus have negative impacts for the poultry industry [2], innovative methods are important for maintaining production. Mesenchymal stem cells (MSCs) seem to provide a suitable tool for examining skeletal development in poultry. MSCs are multipotent cells able to differentiate into osteocytes, chondrocytes, adipocytes, and myocytes [3]. They have been isolated from many species, including chickens, sheep, cats, dogs, rats, mice, and humans [4,5,6,7,8,9,10,11].

MSCs have the capacity to adhere to plastic surfaces under in vitro conditions and to express several surface antigens [12]. They also have the ability to produce cytokines and growth factors, commonly referred to as the MSC secretome, that support and regulate hematopoiesis [13].

MSCs can be used as a model for cell culture to better understand differentiation pathways, as well as to identify supplements that can affect these interactions, and in the areas of viral, skeletal, and immunological research. MSCs have furthermore become a subject of research due to their easy isolation, in vitro proliferation, multi-lineage differentiation, support to hematopoiesis, cytokine and growth factor production, and usefulness for immunomodulatory purposes [14]. Moreover, MSCs are able to migrate through the peripheral circulation to damaged areas, where they proliferate and differentiate, thus facilitating the healing process through the activation of several mechanisms [15]. MSCs are able to reduce cell injury by the synergistic action of small molecules, extracellular vesicles (EVs), secreted by MSCs to maintain tissue homeostasis. Studying the physiological functions of MSCs can improve their application in regenerative medicine and increase our knowledge to better understand their biological behavior [16].

**Figure 1:**
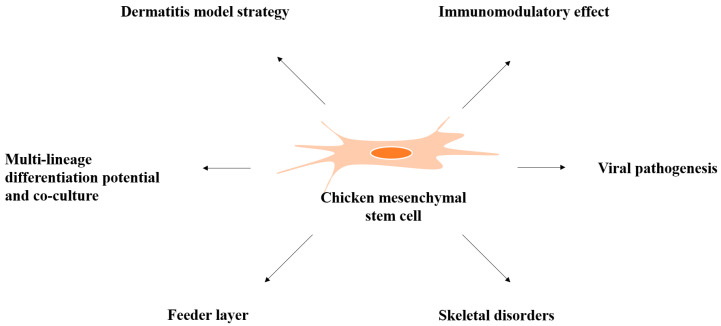
Multifunctional properties of chicken mesenchymal stem cells.

This review covers what is currently known about chicken MSCs. In its first part, we summarize knowledge about their sources, culture conditions, phenotype characterization, and differentiation. In the second part, we focus on the potential for MSCs to be used in three-dimensional (3D) culture and cell-to-cell interactions, their application as a feeder layer, their usefulness for meat production *in vitro*, their cryopreservation possibilities, and their usefulness in researching diseases in chickens (Figure 1).

## 2. Characterization of Chicken MSCs

MSCs commonly are obtained where they were first discovered: from bone marrow as precursors for fibroblasts or stromal cells. An important part of MSC isolation is purification of samples from non-mesenchymal cell types such as hematopoietic and blood cells [17]. MSCs are usually aspirated from bone marrow (in which case they are termed bone marrow-derived MSCs, or BM-MSCs) and then isolated by sieving for plastic adherence in vitro. In addition, MSCs can be obtained from compact bones [18], Wharton’s jelly (WJ-MSCs) [19], and lung (L-MSCs) [20].

The key characteristics defining MSCs have been based on their capacity for colony formation, potential for self-renewal, expression of surface markers, and multi-lineage differentiation. The availability of stem cell-specific markers in poultry has limitations; therefore, researchers have to rely on reports of cell surface markers in mammalian species. Use of markers to verify MSC identity is an important control step to eliminate experimental variability and obtain a homogenous population of MSCs [18]. In mammals, MSCs express surface markers such as CD73, CD90, and CD105 [21,22,23] and transcription factors that include Oct4 [24], Nanog [25], and Sox2 [26], where PouV is a chicken homolog of mammalian Oct4 [27]. MSCs isolated from chicken bone marrow exhibit features similar to those of mammalian MSCs. Furthermore, lung MSCs in chicken and other mammalian species may help in understanding the pathogenesis of infectious and non-infectious lung diseases and the mechanisms of lung injury repair [13]. All chicken MSC characteristic parameters are summarized in the Table 1.

While specific surface markers are easily evaluable, a definition of MSCs can be completed by their abilities to differentiate into classic mesodermal lineages of bone, fat, and cartilage. Because MSCs have the ability to differentiate into osteocytes, chondrocytes, myocytes, and adipocytes, they constitute a suitable and predictable source of cells for purposes of regenerative therapy [30,31,32,33,34]. Supplements such as ascorbic acid and dexamethasone [35] at defined concentrations are able to direct MSCs toward osteogenic differentiation. For chondrogenic differentiation, TGF- β1 often is used as an inducer [36].

The cells are stained using Alizarin Red and Von Kossa (VK) stains for determining mineralization and with alkaline phosphatase (ALP) [35] for detecting osteogenic differentiation at 7 and 14 days of treatment. Moreover, L-MSCs highly express the osteoblast-specific genes OPN and Col-1 in differentiated cells [20]. To stimulate adipogenesis, dexamethasone, indomethacin, insulin, and isobutylmethylxanthine are usually added to the cultures. After 96 h in culture in adipogenic media, Oil Red O is used to determine adipocyte induction. Adipogenic genes such as PPARγ, FABP2, c/EBPα, and c/EBPβ can be used for molecular detection of adipocyte differentiation [18,19]. Hydrocortisone and dexamethasone are well-established inducers of myogenesis. After 72 h, myogenic gene expressions of MyoD, Pax7, Myf5, and myogenin are used in combination with quantitative reverse transcription polymerase chain reaction for detecting myogenesis [18,19]. Vascular endothelial growth factor (VEGF), basic fibroblast growth factor (bFGF), and insulin-like growth factor (IGF-1) are used for endothelial induction, and endothelial markers such as CD34 and CD31 are used for its determination [19]. Moreover, neurogenic differentiation is induced by addition of glial cell-derived neurotrophic factor. Wang et al. (2018) [20] established that nestin and MAP-2, the markers of neural cells, are highly expressed in differentiated L-MSCs. Supplements such as antibiotics and growth factors can affect the phenotypic properties of MSCs and their multi-lineage potential.

MSCs are able to also influence functions of such major immune cells as dendritic cells, T cells, B cells, and natural killer (NK) cells. Although the immunomodulatory mechanism of MSCs is not yet fully understood, there are some mechanisms by which MSCs can be influenced. High levels of pro-inflammatory cytokines can activate MSCs to produce immunosuppressive cytokines, chemokines, as well as nitric oxide (NO) [37]. NO is one of the factors that can suppress T cell proliferation. Moreover, it has been noted that MSCs and macrophages also suppress T cell proliferation via NO inhibition of Stat5 phosphorylation [38,39], and the production of NO during MSC differentiation into chondrocytes has been observed. MSCs have typical in vitro modulatory functions, such as to inhibit T cell and B cell proliferation, as well as DCs differentiation [40,41], thereby resulting in inhibition of immune responses both in vitro and in vivo. Cytokines and growth factors are specific modulators by which MSCs can influence inflammatory responses. This makes MSCs a very promising tool for immunomodulatory cell therapy in immune-mediated diseases. T cells are characterized by the effect of cell proliferation and cytokine secretion. MSC inhibition of T cell proliferation is especially important for immune homeostasis and self-tolerance maintenance [42]. Like mammalian MSCs, chicken MSCs also have immunoregulatory function and inhibit in vitro the mitogenic response of T cells. In chickens, a correlation between NO production and T cell suppression in coculture with MSCs has been noted. Nevertheless, the role of NO in the MSC and T cell coculture system remains unknown.

This review now directs its focus to chickens as a model platform for application of available techniques previously used in other animals that can expand and enrich our knowledge in the area of avian research.

## 3. Biological Properties of Chicken MSCs

### 3.1. Three-Dimensional (3D) Culture and MSC Interactions

Hematopoietic progenitor/stem cells (HPCs/HSCs), MSCs, and multiple elements of the extracellular matrix are components belonging to the microenvironment. It has been noted that MSCs play a crucial role in HPC/HSC function and self-renewal [43,44]. In a study [45] involving the 3D collagen-based culture model, MSCs were used in coculture with HPCs/HSCs. In another study, a significant effect of BM-MSCs upon HPCs/HSCs in terms of self-renewal, maintenance, and differentiation was recorded [46]. BM-MSCs are used as stromal cells for coculture with HPCs/HSCs obtained from umbilical cord blood, and WJ-MSCs are applied as stromal support for HSCs. BM-MSCs in coculture with HPCs/HSCs using the 3D-culture system enable HPC/HSC expansion and regulation of the hematopoiesis process [45]. Study [45] also observed fibronectin production by BM-MSCs to support synthesis of collagen type I and production of osteopontin, which is important for osteogenic differentiation. Moreover, HPCs in the collagen gel containing MSCs revealed initial differentiation into the myeloid lineage, as proven by positivity for CD45. This differentiation was shown by comparison to stromal-cell free conditions as previously described [47].

On the whole, MSCs constitute a promising tool for cellular modulation by secretion and interaction of appropriate molecules to improve regeneration processes in many types of tissues. There are available today many scaffolds, such as micro-/nano- electrospun (EFs) fibers [48,49] or polycaprolactone (PCL) EFs, that modulate paracrine signaling to support cell attachment, proliferation, as well as maintenance of cell stemness and pluripotency [50]. Based on these findings, 3D culture can be used to study the effects of various substances in coculture with MSCs also in chickens in order to better understand the interactions and substitute for the natural environment.

### 3.2. Feeder Cells Layer

Primordial germ cells (PGCs) are progenitors of germ cells, and they have important roles in spermatozoa and egg formation in the adult organism [51]. The most commonly used feeder cells for PGCs culture are xeno-animal buffalo rat liver (BRL) cells, Sandoz inbred mouse-derived thioguanine-resistant and ouabain-resistant (STO) cells, or mouse embryonic fibroblast (MEF) cells, but these cells have their limitations, as contamination of various types can disturb the potential of PGCs. PGC proliferation in vitro depends upon the feeder cells having a powerful capacity to proliferate and secrete cytokines [52,53]. The authors [54] reported that feeder cells can promote proliferation of circulating blood PGCs (cPGCs) and gonadal PGCs (gPGCs) in vitro and that they have the characteristics of an effective feeder layer. Several studies have shown that an MSC-feeder layer ensures all conditions for human-induced pluripotent stem cell (hiPSC), human embryonic stem cell (hESC), and mouse embryonic stem cell (mESC) proliferation and expansion and, moreover, while maintaining cell pluripotency [55,56,57]. MSCs have potential as a feeder cell layer in vitro to provide for expansion of chicken PGCs [54]. Further research is needed to analyze the potential interactions of MSCs via MSC-secreted cytokines with the different chicken cell types.

### 3.3. Infectious Bursal Disease Virus

Poultry are surrounded today by numerous bacterial and viral agents [58]. Infectious bursal disease virus (IBDV), also known as Gumboro disease, was first observed about 60 years ago as an immunosuppressive disorder in young chickens. This virus infects the bursa of Fabricius in young chickens at early ages, and a subclinical form of infection occurs in older birds [59]. The infection leads to morbidity, mortality, and immunosuppression [60]. In vivo presence of IBDV has been detected in several tissues, including bone marrow [61,62]. It is known that MSCs are important cells with the ability to support hematopoiesis and modulate differentiation of hematopoietic stem cells via the expression of cell adhesion molecules necessary for cell-to-cell interactions that result in cytokine and growth factor release [59]. Different types of immune cells are able to modify the host response to IBDV through release of cytokines such as interferon (IFN)- α and IFN- γ [63,64,65,66,67] and pro-inflammatory cytokines such as interleukin (IL)-2 [64,65], IL-18 [65], and IL-6 [65,66]. Inasmuch as the virus does not proliferate in chicken fibroblast cells [68], it therefore can be hypothesized that MSCs could also be a target for IBDV infection. In study [13] they discovered an interaction between IBDV and MSCs. Because MSCs participate in the regulation of hematopoietic precursor differentiation and proliferation, examining these interactions can contribute to better understanding of the virus’s pathogenesis. Moreover, we believe that such stem cells or cell-based vaccines will provide a promising platform or strategy for controlling IBDV and other viruses (zoonoses) with unknown potential risk as a means of preventing potential pandemic diseases.

### 3.4. Skeletal Diseases

Vitamin D3, calcitriol (1,25-(OH)2D3), plays an important role as a nutritional factor relevant to poultry bone strength [69]. Differences between birds and mammals in the formation of the long bones relate to dietary aspects and metabolic activity of 1,25-(OH)2D3. Specifically, avian species have long bone development without secondary ossification until hatching. Ossification only occurs in the proximal and distal ends of the tibiotarsus and tarsometatarsus [70]. The calcium (Ca) level in blood increases the bone strength, and vitamin D facilitates Ca absorption. Therefore, dietary supplementation with adequate Ca and vitamin D is important. It is known that egg laying causes large Ca losses, and so it is necessary to supplement vitamin D to maintain optimal bone structure in laying hens. Tibial dyschondroplasia (TD) is a skeletal disorder in growing chickens characterized by an avascular and non-mineralized growth plate that can lead to deformed tibial bone and lameness [71]. Mineral deficiencies in the diet, and especially insufficient supply of Ca, also can cause keel bone fracture [72,73,74,75,76,77]. Stimulatory effects of 1,25-(OH)2D3 on osteogenic differentiation and mineralization have been recorded in humans [78,79,80,81], in rat osteoblasts [82,83], in mouse osteoblasts [84,85,86,87], and in chicken osteoblasts [88,89]. The amount of an administered substance is important, however, due to the possibility of negative effects. Because MSCs have the abilities for self-renewal and multi-lineage differentiation to osteogenic lineages, they can be used in the study of mineralization during osteogenesis. Vitamin D3 is related to immunoregulation, anti-oxidation, anti-cancer actions, cardiovascular benefits, and such aspects of bone development as osteogenic differentiation and mineralization [89]. Available data suggest that administration of 1,25-(OH)2D3 is important for optimizing bone health in the poultry industry and that avian BM-MSCs constitute a useful tool for examining underlying effects [90]. As we also know, poultry and pig meats have shown the greatest consumption increases [91], and poultry meat consumption has increased in all regions of the world [92].

### 3.5. Probiotics and Prebiotics

Antibiotic growth promoters (AGPs) are widely used in protecting poultry against pathogens and disease and improving growth performance. Prolonged administration of AGPs, however, can lead to bacterial resistance and result in drug residues in poultry products as well as a prohibition against using antibiotics in poultry production. Therefore, it is necessary to develop an alternative pathway for improving production [93].

The chicken gastrointestinal tract is home to a population of microorganisms living in symbiotic relationship with their host, and this relationship is important for the host’s nutrition, metabolism, and immunity—indeed its homeostasis. Although the intestinal microbial environment in adult chickens is highly stable, it can be influenced by feed or stress. One of the major causes of deterioration in meat quality relates to interactions between macronutrients and medications [94].

Live microbial feed supplements known as probiotics, such as *Lactobacillus*, *Bifidobacterium*, or yeasts [95], can confer health benefits to the host. On the other hand, prebiotics, which are non-digestible food ingredients, can enhance lipid metabolism and support polyunsaturated fatty acid levels in chicken meat [96] while resulting in increased levels of health-promoting bacteria within the intestinal tract. Administration of probiotics and prebiotics in feed can improve a flock’s immunity by reducing harmful microbes in the intestine and thus the need for antibiotics [20,93]. Although the efficacy of probiotics and prebiotics in poultry has not yet been sufficiently studied, it is known that MSCs interact with a wide range of intestinal bacteria having significant effects on MSC function [97,98]. MSCs have the ability to home and engraft in the lamina propria of the gastrointestinal tract during intestinal inflammation and exert potent immunomodulatory functions [99]. From this point of view, therefore, it would be pertinent to analyze in vitro interactions between MSCs isolated from AGP-treated animals and applied pro- and prebiotics in order to assess the MSCs profile in terms of, for example, viability, immunomodulatory properties, and values of inflammatory cytokines. With this in mind, the studies would progress to examining the possible effects of pro- and prebiotics on MSC physiology as a possible future replacement for AGPs.

### 3.6. Chicken Dermatitis

Gangrenous dermatitis (GD) is a disorder that affects broiler chickens and results in economic losses in the poultry industry worldwide [100]. GD is primarily caused by the Gram-positive anaerobic bacilli *Clostridium perfringens* type A [101,102,103,104], *Clostridium septicum*, and *Staphylococcus aureus* [105]. The disease is characterized by hemorrhage, congestion, and necrosis of the skin as indicated by edema. The breast, abdomen, back, thighs, tail, and wings are the most significantly affected body areas [106]. The dermatitis results in lower meat quality [107]. Typical symptoms of GD are poor appetite, decreased muscle coordination, skin edema, leg weakness, and ultimately crepitus [108]. GD in chickens causes decreased splenocyte proliferation in response to concanavalin A (Con A) or lipopolysaccharide (LPS); greater levels of serum NO and a-1-acid glycoprotein (a-1-AGP); higher levels of T cells, B cells, and macrophages; as well as increased levels of transcripts encoding IL-8, IFN-a, TNFSF-15, and LITAF compared with GD-free chickens [94]. Therefore, MSCs can be useful in evaluating a variety of diseases, including mainly tissue-related and immune-mediated diseases, due to their ability to modulate the innate and adaptive immune systems. Several experimental models have been used to clarify the role of *C. septicum*, *C. perfringens*, and *S. aureus* in the pathogenesis of GD in vivo [109], but MSC coculture in vitro with the aforementioned pathogens can be a promising approach for assessing their interactions as well as their immunomodulatory properties.

### 3.7. Meat “In Vitro”

Nowadays, meat production is the major source of pollution. World meat production contributes between 15 and 24% of greenhouse gas emissions [110]. It is known that chicken meat production requires 3, 918m^3^/ton of water [111]. Therefore, satisfying the demand for meat in the future will be a challenge when we intend on maximizing the use of agricultural sources and reducing the greenhouse gas production. Meat worldwide consumption was calculated by Fiala [112], who predicts a 72% increase in meat production in 2030 compared to 2000. The 1918 H1N1 Spanish Influenza pandemic [113] as well as SARS-CoV-2 are of utmost concern as they have spread to almost all countries and killed thousands of people worldwide [114]. The correlation between meat production and outbreaks of diseases cannot be overlooked.

**Figure 2:**
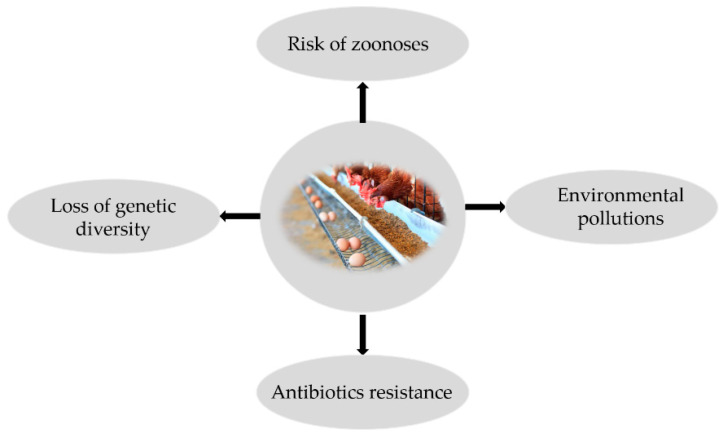
Disadvantages of commercial farming of livestock.

In vitro meat culturing seems to be a suitable substitution for conventional meat production. In vitro cultured meat from stem cells in controlled culture and physiological conditions in the laboratory uses MSCs that are able to differentiate to myocyte or induced pluripotent stem cells (iPSCs) by genome reprogramming of somatic cells [115]. In vitro meat culturing has many advantages: (a) in vitro cultivation is faster than growth, (b) the impact of cultivation on the environment is lower, and (c) muscle tissue is cultivated without affecting the skeleton [116]; moreover, cultured meat is a healthier, cleaner, and disease-free animal protein source compared to commercial farming [117] (Figure 2).

Cell culture also involves scaffold-based cell and tissue approaches relying on the isolation, culture, and differentiation into myoblasts. However, there is possibility to coculture stem cells with adipocytes, which support them to differentiate, and then form myofibers [118]. However, cell culture requires many growth factors such as IGF, bFGF, HGF, Wnt3a, and Wnt7a to promote differentiation into myotubes and myofibers [119]. Moreover, scaffolds have an important role in the cell culture-based approach for in vitro meat production. Scaffolds are commonly made of natural and edible polymeric biomaterials such as collagen that allow 3D tissue culture and subsequent complex structuring of synthetic meat [120,121].

We believe that this transformation in the farm process will be inevitable. Moreover, this technology offers an opportunity for non-meat eaters because this meat is safe and free from animal slaughtering and cruelty. Since in vitro production is controlled, it is feasible to alter high-quality meat production on a sustainable basis. From this point of view, extra embryonic and adult MSCs could be navigated toward myocyte differentiation, which ultimately forms myotubes and are, thus, suggested as a potential starting point for in vitro chicken meat production.

### 3.8. Cryopreservation

In recent years, several animal stem cell banks have been established worldwide. Although these banks are not used for therapeutic purposes, they are considered a promising way for the storage of animal genetic resources [108]. For this reason, we have noticed the increased demand for national gene banks to preserve either native or worldwide biodiversity. These banks preserve genetic information from many important (already endangered) livestock species. However, in addition to embryos and gametes, adult stem cells also represent a significant genetic resource that can be obtained from various biological sources.

The stem cell banks also give a source of stem cell lines that have high quality and safety standards [122]. Many animal embryonic and adult stem cell lines have been made and cryopreserved, including primordial, bone marrow mesenchymal, neural, cardiac, endothelial, adipose, and umbilical cord mesenchymal stem cells [123,124]. Biological research could use the method of cryopreservation of stem cells in other fields as well, as it has already given promising results [123]. Cryopreservation of stem cells is important to provide storage of high cell numbers, fast transport, and to preserve cells for long periods. Due to the increased level of endangered animals, it is important to preserve genetic material for future applications.

The chicken was the first farm animal with a completely sequenced genome. Because of its in ovo embryonic development rather than in utero, the chicken is a suitable model for embryology and development studies. The chicken provides a model organism for the study of cancer and viruses. The first tumor virus and oncogene (src), Rous sarcoma virus, was identified in the chicken. The immune system of chickens provides the first indication of the distinctions between T and B cells, with the B-cell based avian bursa of Fabricius [125]. Therefore, the chicken is an important model for evolution, embryology, cell biology, immunology, virology, oncology, and gene regulation studies. From this point of view, it is also important to cryopreserve MSCs for subsequent assessment of MSCs in vitro and further usage in the future (e.g., iPSCs).

## 4. Conclusions

This review has emphasized the importance of chicken MSCs for their self-renewal potential and multi-lineage differentiation as well as current knowledge concerning their usefulness for examining pathogenic potential of infectious bursal disease virus, studying mineralization during osteogenesis, and using MSCs as a feeder layer. MSCs also constitute a very promising tool for immunomodulatory cell therapy in immune-mediated diseases due to their inhibition effect on T cell proliferation via NO production. Moreover, MSC-based treatment could be a model strategy in studying chicken dermatitis disorders as well as for reducing the need to administer AGPs. Next, BM-MSCs in coculture with hematopoietic progenitor/stem cells can provide expansion and regulation of the hematopoiesis process using the 3D-culture system for future research in chickens. Finally, current meat production methods are associated with many problems such as animal welfare issues, risk of infectious diseases, biodiversity loss, and environmental pollution. Therefore, MSCs provide an alternative way to eliminate these problems using in vitro meat culturing. Based on these findings, we may conclude that MSCs can provide a useful model in the field of chicken stem cell research.

## Figures and Tables

**Table 1:** Comparison of chicken MSCs derived from different sources.

Sources	Type of Digestive	Separation	Morphology	Confluency	Positive Markers	Negative Markers	Transcription Factors	References
Bone marrow	-	Ficoll-Hypaque (1.090 g/mL)	spindle-shaped	14 days	CD44, CD90 CD105	CD45	PouV, Sox2, Nanog	[17]
		Percoll solution (1.073 g/mL)	spindle-shaped	2–3 days	CD44, CD29, CD71, CD73	CD31, CD34		[19]
Compact bones	0.25% collagenase		spindle-shaped	8–10 days	CD90, CD105, CD73, CD44	CD31, CD34 CD45		[18]
Lung	0.1% collagenase		spindle-shaped	5–7 days	CD29, CD73, CD90, CD105	CD34, CD45	OCT-4	[20]
	0.5 mg/mL collagenase type IV	Ficoll-Hypaque (1.090 g/mL)	spindle-shaped	14 days	CD44, CD90, CD105		PouV	[28]
Wharton’s jelly	0.1% collagenase type IV		fibroblast-like shaped	5–6 days	CD29, CD44, CD71, CD73	CD31, CD34		[29]

## Data Availability

No new data were created or analyzed in this study.
